# Tailoring pore distribution in polymer films *via* evaporation induced phase separation†

**DOI:** 10.1039/c9ra01331h

**Published:** 2019-05-17

**Authors:** Rumiaya Pervin, Pijush Ghosh, Madivala G. Basavaraj

**Affiliations:** Department of Chemical Engineering, Indian Institute of Technology Madras Chennai 600036 India basa@iitm.ac.in; Department of Applied Mechanics, Indian Institute of Technology Madras Chennai 600036 India

## Abstract

By considering a mixture of poly(methyl methacrylate)–tetrahydrofuran–water (PMMA–THF–H_2_O), we report an experimental approach to tune the distribution of pores in polymer films formed *via* evaporation induced phase separation (EIPS). We show that the drying induced composition and microstructural changes that occur due to the evaporation of the solvent (THF) and the nonsolvent (H_2_O) delineate the ultimate polymer film morphology. The temporal evolution of the microstructure, the phase behavior and the change in the composition of the PMMA–THF–H_2_O mixture at air–solution (top surface) and solution–substrate (bottom surface) interfaces is monitored to provide insights into the origin of the pore distribution in the final polymer films. The effects of various parameters such as nonsolvent and polymer concentration in the casting solution, casting solution thickness, relative humidity and temperature on the final film morphology are investigated to correlate how the composition path (CP) change under various conditions ultimately dictates the film morphology. We show that depending on the change in the composition of the polymer solution (evolution of CP) and the water/PMMA ratio at the time of phase separation, the morphology of the final film formed varies as – (1) non porous *i.e.*, dense film (2) a film with pores only at the bottom surface (3) an asymmetric film *i.e.*, films with a top dense layer (non-porous) supported by porous sub-layers (4) a porous film with uniform pores distributed across the entire film thickness and (5) a film with pores only at the top surface. In addition, we show that the morphology of the PMMA film can also be tuned by varying the composition of low and high molecular weight PMMA in the casting solution. These porous PMMA structures, being biocompatible, are useful for applications in cell culture, drug delivery and wound dressing.

## Abstract 2

The work contributes to a fundamental understanding of how to regulate the microstructure of porous polymer films fabricated *via* evaporation induced phase separation (EIPS).

## Graphical abstract

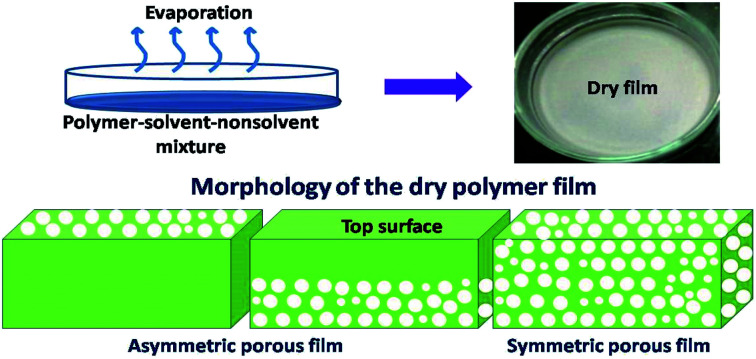

## Introduction

Polymeric films of controlled pore distribution with either asymmetric (nonporous top layer supported by porous sub layers) or symmetric (pores distributed throughout the film thickness) pore morphology are promising materials for various applications as controlled release systems, wound healing scaffolds, ultra-filtration membranes or as breathable fabrics.^1–7^ The liquid–liquid phase separation (LLPS) route is undoubtedly a well-known and established technique to prepare porous polymeric structures.^8–16^ In this process, a thermodynamically unstable polymer solution phase separates into two coexisting liquid phases in thermodynamic equilibrium. One of these phases, *i.e.*, the polymer-rich phase solidifies to form a solid matrix, while the other phase, *viz.*, a polymer-lean phase which is rich in nonsolvent, eventually forms the pores in the matrix. The LLPS can be realized by several routes – wet cast phase separation (WCPS) or immersion precipitation technique (IPT), evaporation induced phase separation (EIPS) or dry cast phase separation (DCPS), vapor induced phase separation (VIPS), non-solvent induced phase separation (NIPS), reaction induced phase separation (RIPS) and thermally induced phase separation (TIPS). In these techniques, the thermodynamic instability in a polymer solution that eventually leads to LLPS is induced by varying certain parameters such that the composition of the polymer solution approaches the vicinity of the binodal curve. In evaporation induced phase separation technique (EIPS), the liquid–liquid phase separation is achieved by controlled evaporation (temperature, relative humidity) of the solvent and the nonsolvent from an initially homogeneous single-phase polymer solution. Although EIPS is a much simpler process in terms of cost, time, ease and the control that it offers over the final film morphology, it is comparatively less explored compared to other phase separation methods.^12,17^ EIPS has been used for the production of polymer films of controlled porosity with asymmetric and symmetric pore morphology.

The EIPS technique also called the dry casting process was pioneered by Kesting^8^ in 1973. Jansen *et al.*^6^ used this method for the formation of asymmetric poly(ether ether ketone) membranes by using various solvent–nonsolvent combinations for applications in gas separation. EIPS method has been used to demonstrate the fabrication of densely packed micro patterned poly(methyl methacrylate) and polystyrene surfaces on different substrates^3,4^ for the study of cell culture. Shojaie *et al.*^18^ described a theoretical model which incorporates coupled heat and mass transfer where self and main diffusion coefficients of the evaporating species are included to describe the evaporation of both solvent and nonsolvent from a homogeneous polymer–solvent–nonsolvent system to understand the membrane morphology obtained by EIPS. This approach enabled correlating the final film morphology with theoretical prediction of the evolution of composition of the casting solution during drying *i.e.*, the composition path (CP). In addition, they studied membrane morphology for cellulose acetate–acetone–water system to validate their model. Matsuyama *et al.*^19^ proposed mathematical model to elucidate the membrane morphology obtained by dry casting technique from cellulose acetate–acetone–nonsolvent system. In their study, several high boiling point nonsolvents are used to ensure that the composition change occurs solely due to the evaporation of the solvent. Although, the model considers mass transfer process and the change in polymer volume fractions during the membrane formation, only self diffusion coefficients are incorporated in the analysis. The effects of the weight fractions of nonsolvent and polymer, casting solution thickness and different types of nonsolvents on the membrane morphology are investigated by predicting CP from the model. Altinkaya *et al.*^17^ developed a mathematical model in which the ternary diffusivities *i.e.*, self, main and cross diffusion coefficients of the evaporating species are incorporated to predict the morphology of the polymer films typically generated by this technique. The predictions of CP from the model are used to explain the microstructure of the polymer films generated from cellulose acetate–acetone–water ternary mixture under various conditions such as polymer/nonsolvent ratio, initial film thickness, and temperature, relative humidity and air velocity.

The predictions of CP from the theoretical models discussed so far suggest that the formation of asymmetric polymer films is due to markedly different composition of the casting solution at the air–solution (top surface) and solution–substrate (bottom surface) interface at the point of phase separation. Surprisingly, in spite of the fact that CP for both the air facing (casting solution–air interface) and substrate facing (casting solution–solid interface) surface predicted from the theoretical models cross the phase boundary, the polymer films formed possess asymmetric porous structure *i.e.*, films are partly porous and partly non-porous. This is thermodynamically not feasible; as such composition paths are expected to lead to polymer films that contain pores throughout the film (symmetric pores). The formation of asymmetric films in these studies^17,18^ is attributed to the difference in polymer volume fraction between the top surface and the bottom surface at the moment of phase separation. The difference in the polymer volume fraction can not solely explain the asymmetry in the final polymer film as there still exists a difference in the polymer volume even in case of symmetric and completely dense films obtained in their studies. This demands an investigation of the composition path of the casting solution at the air–solution (top surface) and solution–substrate (bottom surface) interface during drying.

In this article, PMMA–THF–H_2_O ternary system is considered for the fabrication of porous films of controlled morphology *via* the evaporation induced phase separation. The interest in the fabrication and wide spread application of poly(methyl methacrylate) (PMMA) films is due its biocompatibility, temperature resistance, excellent weather ability, good optical property, and cost-effectiveness. PMMA films being versatile find applications in several areas such as packaging, biomedical, optics, thermal insulation, adsorption and membrane separation technologies. We experimentally analyze the evolution of the microstructure and the change in composition of the PMMA–THF–H_2_O casting solution close to the air–solution and solution–substrate interface of the casting solution during the fabrication of the polymer films. We construct ternary isothermal phase diagram of polymer–solvent–nonsolvent system by the cloud point measurement. The composition path measured by monitoring the conductivity of the casting solution during drying is plotted on the phase diagram to comprehend the phase behavior during film formation, the corresponding microstructure of the casting solution and the final polymer film morphology. The effects of various parameters such as nonsolvent and polymer concentration in the casting solution, casting solution thickness, relative humidity and temperature on the final film morphology is studied to correlate how the composition path change under various conditions ultimately dictates the film morphology. We also studied the effect of molecular weight and polydispersity index on the morphology of the PMMA films. We show that both thermodynamic and kinetic parameters affect the pore distribution in the dry polymer films. Depending on the change in composition of the polymer solution (evolution of CP) under various conditions, the morphology of the final film formed varies from non porous (*i.e.*, dense) to porous with asymmetric or symmetric pore morphology.

## Methods section

### Reagents

Poly(methyl methacrylate) (PMMA) of 120 000 g mol^−1^ and 15 000 g mol^−1^ weight average molecular weight was used for the fabrication of polymer films. PMMA and anhydrous THF (tetrahydrofuran) which is a good solvent for PMMA (≥99.9% purity) was purchased from Sigma Aldrich, USA and used as received. The boiling point of THF is 66 °C. In all the experiments, de-ionized water (which is a non-solvent for PMMA) obtained from Milli-Q system (Merck Millipore) was used.

### Phase diagram of PMMA–THF–H_2_O ternary system

The bulk phase behavior of PMMA–THF–H_2_O mixture was studied to understand the onset of phase separation and its relation to the final morphology of the dry polymer film. The ternary isothermal phase diagram was determined experimentally by visual observation of the appearance of turbidity in the mixture. A predetermined quantity of PMMA–THF solution (0.5 to 40 wt/wt% PMMA) was taken in the clean vial covered with Teflon tape (to prevent evaporation of volatile THF) and water was added dropwise till the clear homogeneous solution visually turns turbid. During the addition of water, the solution was continuously stirred and maintained at a constant 30 °C temperature. The water concentration at which turbidity appears was recorded. The amount of water added was measured by an analytical balance. After the appearance of turbidity, the addition of water was stopped and the solution was stirred further to check the persistence of turbidity. If the solution turns clear, water was further added and the same procedure was repeated. From the quantity of water that leads to turbid solution, the concentration of nonsolvent, solvent and polymer in the vial was estimated and the composition corresponding to this is termed as the cloud point. The same procedure was followed for different solutions of different PMMA–THF concentrations. The plot obtained from the cloud points measurement that separates the stable single phase region from the unstable two phase region in the ternary phase diagram is known as the binodal curve. The maximum concentration of PMMA in the initial PMMA–THF solution was limited to 40 wt%. At higher polymer concentration, the polymer solution was inherently turbid; moreover the viscosity was too high to obtain a homogeneous solution. At the cloud point, the homogeneous solution separates into two phases-sol *i.e.*, polymer lean phase and gel *i.e.*, polymer rich phase. The concentration of polymer at which the volume of polymer lean phase is equal to the polymer rich phase is known as critical concentration and this point is called the critical point. By measuring the equilibrium volume of the two phases (polymer lean and polymer rich phase) at the cloud point (details given in the ESI, Fig. SI 1†), the critical point was obtained. In order to establish critical point, the two-phase mixture at cloud point was sealed in a vial and kept undisturbed until thermodynamic equilibrium was achieved. At equilibrium, both the phases formed were transparent and the volumes of the coexisting phases were determined.

### Morphology of the PMMA–THF–H_2_O casting solution during drying

A homogeneous solution of three components PMMA–THF–H_2_O was obtained by dissolving a known quantity of PMMA (5, 10 and 15 wt/wt%) in THF followed by dropwise addition of water (0, 1, 2, 3 and 4 wt%) under constant stirring. This procedure resulted in a transparent homogeneous PMMA–THF–H_2_O casting solution of known composition. Typically, 5.5 ml PMMA–THF–H_2_O mixture taken in a 5 cm diameter glass Petri dish was evaporated at 30 °C temperature and 60% relative humidity (RH). The morphological change as a consequence of evaporation of solvent and nonsolvent during the drying process was recorded with an inverted optical microscope (Leica DMA 3000 B, Germany). The images were captured at magnification of 10 and 20×. A digital camera was used to obtain images of the visual appearance of the sample during drying.

### Preparation of polymer film

Several polymer films were synthesized by considering casting solutions of various initial compositions and experimental conditions as mentioned in the Table 1. A homogeneous three component PMMA–THF–water solution was prepared as discussed in Section 2.3. The solution was then casted in a 5 cm diameter glass Petri dish. The complete evaporation of THF and water from the casting solution resulted in the formation of PMMA film. In all the experiments the thickness of the casting solution was 5 mm, the volume of the PMMA–THF–water solution used was 5.5 ml. While most of the experiments were conducted at constant temperature of 30 °C and 60% RH, some experiments were also performed at 50 °C and 74% RH. The effect of temperature, casting solution thickness, relative humidity was also investigated, the details of which can be found in Table 1. The prepared films were stored under dry environment in a dessicator until further analysis.

**Table 1:** The composition of water, THF and PMMA in the homogeneous casting solution used for the fabrication of polymer films and details of the experimental conditions

Sample ID	wt/wt%			Casting solution thickness	Temperature (°C)	Relative humidity (%)	Average porosity (%)	Film morphology	Average pore diameter (μm)
	Water	THF	PMMA						
**The effect of water (1–4 wt%) concentration while other variables such as PMMA (5 wt%), solution thickness (5 mm), temperature (30 °C) and relative humidity (60%) were constant**									
S_1W5P_	1	94	5	5	30	60	—	Nonporous	—
S_2W5P_	2	93	5	5	30	60	10.3 ± 0.62	Bottom surface pore	3.5 ± 0.45
S_3W5P_	3	92	5	5	30	60	29.3 ± 0.84	Asymmetric porous film (29% thick dense layer)	7.05 ± 2.67
S_4W5P_	4	91	5	5	30	60	36.9 ± 0.72	Symmetric porous	2.28 ± 0.52
**The effect of PMMA (5–15 wt%) concentration while other variables such as water (4 wt%), solution thickness (5 mm), temperature (30 °C) and relative humidity (60%) were constant**									
S_4W5P_	4	91	5	5	30	60	36.9 ± 0.72	Symmetric porous	2.28 ± 0.52
S_4W10P_	4	86	10	5	30	60	17.4 ± 0.42	Asymmetric porous film (66.9% thick dense layer)	3.43 ± 0.13
S_4W15P_	4	76	15	5	30	60	9 ± 0.47	Bottom surface pore	2.67 ± 0.27
**The influence of casting solution thickness (5 mm and 1 mm) while the concentration of water (3 wt%), PMMA (5 wt%), temperature (30 °C) and relative humidity (60%) were constant**									
S_3W5P_	3	92	5	5	30	60	29.3 ± 0.84	Asymmetric porous film (29% thick dense layer)	7.05 ± 2.67
S_3W5PTh_	3	92	5	1	30	60	22.3 ± 0.64	Asymmetric porous film (87.82% thick dense layer)	3.13 ± 0.42
**The effect of temperature (30 °C and 50 °C) while the concentration of water (2, 3 and 4 wt%), PMMA (5 wt%), solution thickness (5 mm) and temperature (30 °C) were constant**									
S_2W5P_	2	93	5	5	30	60	10.3 ± 0.62	Bottom surface pore	3.5 ± 0.45
S_2W5PT_	2	93	5	5	50	60	—	Nonporous	—
S_3W5P_	3	92	5	5	30	60	29.3 ± 0.84	Asymmetric porous film (29% thick dense layer)	7.05 ± 2.67
S_3W5PT_	3	92	5	5	50	60	—	Nonporous	—
S_4W5P_	4	91	5	5	30	60	36.9 ± 0.72	Symmetric porous	2.28 ± 0.52
S_4W5PT_	4	91	5	5	50	60	10.7 ± 0.76	Top surface pore	4.52 ± 0.54
**The effect of relative humidity (60% and 74%) while the concentration of water (0 and 1 wt%), PMMA (5 wt%), solution thickness (5 mm) and temperature (30 °C) were constant**									
S_1W5P_	1	94	5	5	30	60	—	Nonporous	—
S_0W5PH_	0	95	5	5	30	74	12.3 ± 0.42	Top surface pore	3.3 ± 0.21
S_1W5PH_	1	94	5	5	30	74	14.3 ± 0.73	Top surface pore	3.15 ± 0.52

The subscripts W, P, Th, T and H used in the sample IDs stand for water concentration, PMMA concentration, casting solution thickness, temperature and relative humidity respectively. The numbers mentioned before the subscripts W and P correspond to the concentration of water and PMMA in wt%. For example, sample ID denoted by S_1W5P_ represents casting solution containing 1 wt% water and 5 wt% PMMA. Note that some samples IDs do not have the subscripts Th, T and H. In such cases, all experiments were conducted at constant casting solution thickness (Th = 5 mm), temperature (*T* = 30 °C) and relative humidity (*H* = 60%). The presence of subscript Th in the sample ID refers to the use a casting solution of 1 mm thickness. Similarly, the subscript T and H in the sample IDs refer to casting solutions dried at 50 °C and 74% RH respectively.

### Characterization of polymer film

The morphological features of the films were characterized with a high resolution scanning electron microscope (SEM) (S-4800, Hitachi®) under an accelerating voltage of 1–3 kV. The sample specimens were sputter coated with a thin gold film for 60 s using an ion coater (Hitachi E-1300) prior to SEM observation. Small sections of the film were used to image the morphology of the film surface in contact with the Petri dish and that exposed to air. In order to obtain cross-sectional view, the dried films were sectioned by a microtome (Leica EM UC7, Germany). During microtome an initial cutting speed of 2 mm s^−1^ and cutting depth of 1 μm were used and towards the end, a speed of 1 mm s^−1^ with cutting depth of 500 nm were maintained in order to obtain smooth surfaces. The thickness of each film was directly measured from the cross sectional SEM images and by multiple point digital micrometer. The mass of the film, *m*, was measured by an analytical balance. The overall porosity (*ε*) or void fraction was calculated from the thickness *l*, the area *A* and the mass *m* of the polymer film samples:^6^

1 *ε*(%) = (*V*_v_/*V*_t_) × 100 = (1 − *V*_p_/*V*_t_) × 100 = (1 − *m*/(*ρlA*)) × 100

where *V*_v_, *V*_t_ and *V*_p_ are the void volume, the volume of the polymer film and the volume of polymer in the film respectively and *ρ* is the density of PMMA which is equal to 1.18 g cm^−3^. The average pore diameter was measured from the SEM images of the surface of the films by ImageJ software.

### FTIR and XRD analysis of PMMA films

The dry PMMA films were further characterized by Fourier-transform infrared spectroscopy (FTIR) and X-ray diffraction (XRD). A FTIR spectrometer (Cary 630, Agilent) with 4 cm^−1^ resolution was used to study the characteristic absorption peak corresponding to different functional group in the PMMA films in the wavenumber range from 3500 cm^−1^ to 400 cm^−1^. The FTIR and XRD spectra for non porous and porous PMMA films of different morphologies were recorded. A Bruker D8 Discover powder XRD diffractometer was used for analyzing the crystallinity of PMMA powder and the dry PMMA films in the 2*θ* range 10–90°. A small quantity of PMMA powder was placed on a glass slide for XRD analysis. For FTIR and XRD analysis, a small piece of the of PMMA film cut in the form of a square of 5 × 5 mm^2^ area was used.

### Determination of composition of the PMMA–THF–H_2_O solution during evaporation

The change in the composition of the PMMA–THF–H_2_O casting solution during drying was determined by conductivity measurement. As the weight of polymer remains constant and the weight loss is solely due to THF and water evaporation, the total weight of THF–water present in the casting solution at any time instant was measured using a microbalance. However, the concentration of each component cannot be determined. Therefore, weight change measurements were combined with the measurement of conductivity of the casting solution. The conductivity of water used in the preparation of casting solution was in the range of 0.5–0.6 μS cm^−1^ due to presence of small quantity dissolved ions. As PMMA and THF do not contribute to solution conductivity, the change in the conductivity of PMMA–THF–H_2_O solution during film formation is dictated solely by the change in water concentration and the dissolved ions in the solution. In conductivity experiments, a CM-230 conductivity monitor that can measure conductivity in 0.1–2000 μS cm^−1^ range at 0.1 μS cm^−1^ resolution was used. The conductivity probe consists of Teflon coated 1 mm diameter aluminium rod electrodes constructed in-house. The cylindrical surfaces of the electrodes were coated entirely with the Teflon except for the bottom circular surface (details given in ESI†). This enables the measurement of conductivity of a solution at any depth. The conductivity meter was calibrated using a standard sodium chloride (NaCl) electrolyte solution. The cell constant, determined based on the conductivity of 0.84 mM aqueous NaCl solution (100 μS cm^−1^) and standard solution (1413 μS cm^−1^) supplied by HAANA was 1.4 cm^−1^. Approximately, 5.5 ml of solution was taken in a 5 cm diameter Petri dish and the probe was positioned at the top and bottom surface of the solution in two separate experiments and the conductivity was recorded.

The conductivity was measured for THF, water and mixture of THF–water (with different concentrations) at 25 °C and 30 °C. A calibration curve (Fig. SI 4, in ESI†) was obtained by plotting the conductivity as a function of the mole percent of water in the mixture. The weight of the THF–water mixture in the casting solution was measured by an analytical balance with accuracy up to four decimal places. From the calibration curve and the weight of the casting solution at each time instant, the concentration of each component in the casting solution at a given time was measured. The change in composition at each step was plotted in the phase diagram to obtain the composition path that the casting solution undergoes during the course of film formation. The data were collected when there was a change in conductivity of 0.1 μS cm^−1^. In the conductivity cum weight measurement method used for measuring the composition path, we assumed that the mass of polymer at the air–solution and the solution–substrate interface was same as the mass of polymer in the casting solution at time, *t* = 0 as long as the solution remains homogeneous and single phase.

## Results and discussion

### Ternary phase diagram of PMMA–THF–H_2_O solution

**Fig. 1:**
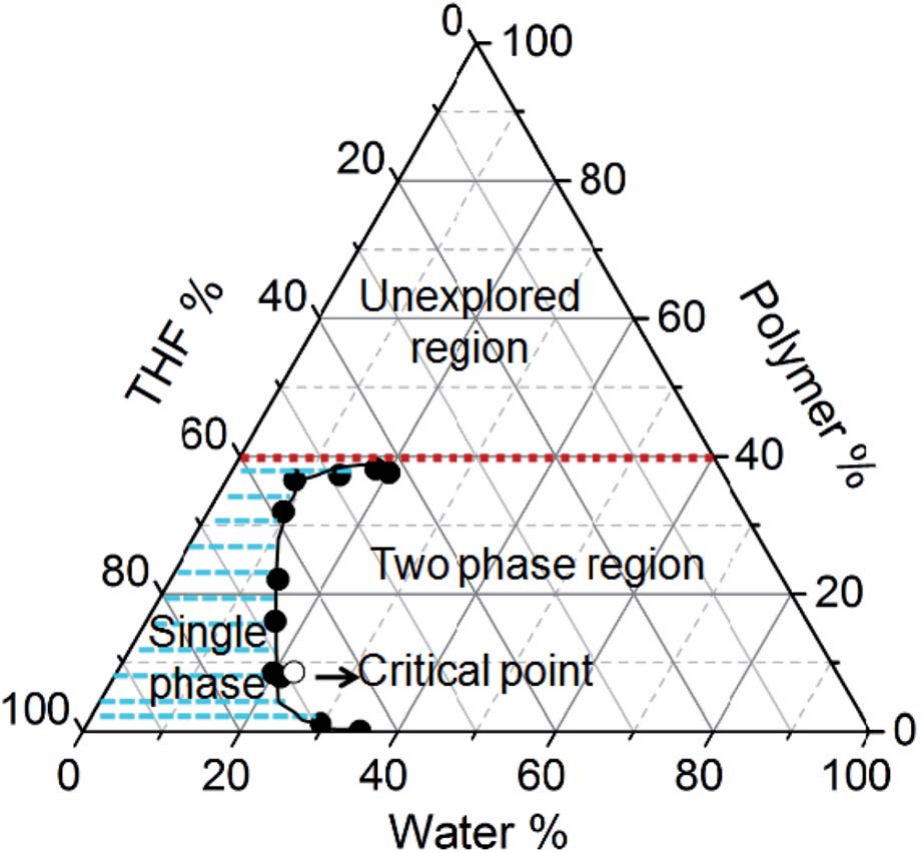
The phase diagram of PMMA–THF–H_2_O system at 30 °C. The solid and open circle respectively represents the cloud-points and the critical point. The solid line connecting the cloud points is the binodal curve. The shaded region is a single phase region and the region to the right of the binodal curve represents the two phase region. The region above the horizontal dotted red line indicates samples with polymer concentrations above 40% for which the cloud points could not be determined experimentally due to highly viscous nature.

Fig. 1 shows the phase diagram of the PMMA–THF–H_2_O ternary mixture that was obtained by the cloud point measurement, which involved identifying the state of the mixture of different, but known compositions. The details of the cloud point measurement are given in ESI Fig. SI 1.† The phase diagram was established experimentally at a constant temperature of 30 °C. In Fig. 1, the vertex of the triangle represents a pure component; a point along the side of the triangle represents a mixture of two components and any point inside the triangle corresponds to a three component mixture. The shaded region in Fig. 1 represents the single phase region. The solid circles in the diagram represent the cloud points that separate the two-phase region from the solutions that are homogeneous single phase. The solid line connecting the cloud points is called the binodal curve and the region to the right of this curve represents the two phase region. The open circle symbol in Fig. 1 depicts the critical point, defined as the composition at which the volume of the polymer rich is equal to the polymer lean phase at equilibrium. The concentration of polymer at this point is called as critical concentration (Fig. SI 1 in ESI†). In our study, the critical point was obtained at 8.6, 69.1 and 22.3 wt% of PMMA, THF and water respectively at 30 °C. Schuhmacher *et al.*^7^ reported the critical point as 9.8 wt% PMMA, 74.0 wt% THF and 16.2 wt% water at 25 °C. The microstructures of the ternary mixture of corresponding to different compositions in the phase diagram reveal different morphologies shown in Fig. SI 2 in the ESI.† These morphologies provide a broad idea about the composition of the casting solution that must be considered for the fabrication of the porous polymer films.

### Time resolved macro and microstructural changes during film formation

**Fig. 2:**
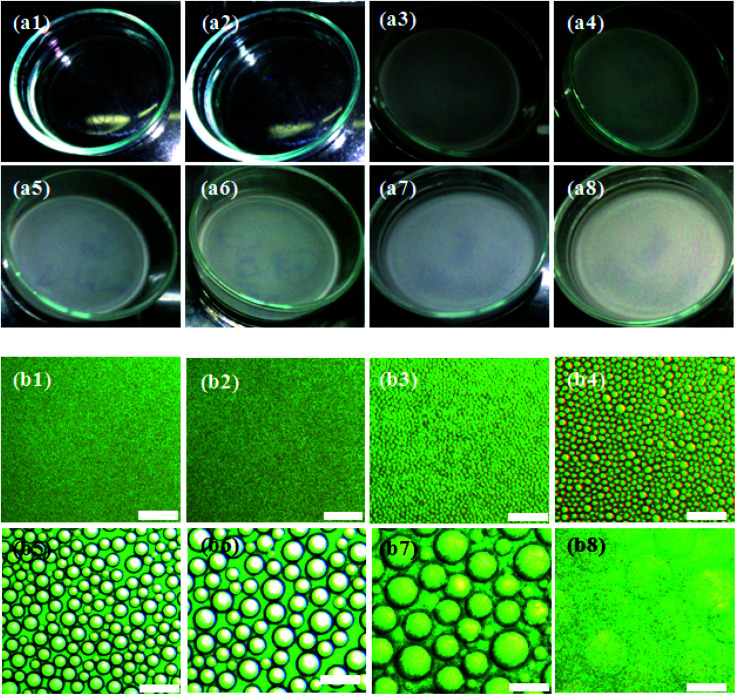
Characterization of the morphology of the PMMA–THF–water homogeneous solution corresponding to sample S_4W5P_ during the evaporation of solvent and nonsolvent: (a1 to a8) are the digital camera images of the solution in 5 cm diameter Petri dish at (a1) 0, (a2) 48, (a3) 51, (a4) 52, (a5) 53, (a6) 54, (a7) 55 and (a8) 57 min. The microstructure of the polymer solution imaged by an optical microscopy at the corresponding time interval is shown in (b1 to b8). The scale bar of each image in (b1 to b8) is 2 μm. The water droplets nucleate at *t* = 51 min due to liquid–liquid phase separation as shown in (b3) and further grow in size due to coalescence. Interestingly, the appearance of turbidity in (a3) coincides with the onset of phase separation in (b3).

Investigating the evolution of the microstructure of the ternary casting solution facilitates an understanding of the phase separation mechanism and pore nucleation that eventually governs the distribution of pores in the final film. The homogeneous polymer solutions S_1W5P_, S_2W5P_, S_3W5P_ and S_4W5P_ prepared by dissolving 5 wt% PMMA in THF and subsequent addition of 1, 2, 3 and 4 wt% of water were visibly transparent and single phase. Fig. 2 shows the digital camera images of the casting solution S_4W5P_ and the corresponding optical microscopy images at different time during drying. As shown in Fig. 2, 48 min into the film formation process, the casting solution still remains transparent and single phase. As evident in Fig. 2(a3), at *t* = 51 min, the casting solution turns turbid. This co-inside with the formation of spherical drops as evident in the corresponding microscopic image in Fig. 2(b3). This suggests that the polymer solution undergoes liquid–liquid phase separation by nucleation and growth mechanism and thus droplets of minority phase *i.e.* water appear in the casting solution. After nucleation, the water droplets were observed to grow at a faster rate than that typically observed during Ostwald ripening (Fig. SI 3, in ESI†). This suggests the growth of drops *via* coalescence. The latter is expected at the final stage of phase separation. Moreover, Ostwald ripening occurs in very dilute solution when the concentration of the non-solvent (water) tends to zero. In Ostwald ripening, the diffusional mass exchange leads to growth of bigger droplets at the expense (or disappearance) of smaller droplets.^20–23^ The nucleation of water droplets at 60, 55 and 51 min respectively for polymer solutions containing 2 (S_2W5P_), 3 (S_3W5P_) and 4 (S_4W5P_) wt% water was observed to coincide with the appearance of cloudiness in the casting solution. Thereafter, the casting solution turns turbid similar to the observations in Fig. 2(a1 to a8) and the intensity of turbidity increases with time. Finally, an opaque polymer film was obtained. When sample S_1W5P_ is dried, in spite of the presence of 1 wt% water, the casting solution remained transparent throughout the drying process and there were no changes in the microstructure of the solution. Therefore, not just the presence of water, but the occurrence liquid–liquid phase separation is crucial for the formation of pores in the final polymer films. The porous structures achieved by liquid–liquid phase separation and solidification have been reported for PLA (polylacticacid),^24^ PEEK (poly ether ether ketone),^6^ cellulose acetate,^2^ PI (polyimide).^25^ The results discussed so far reveal that porous microstructure of the polymer film is due to nucleation and growth of nonsolvent droplet through liquid–liquid phase separation. As elucidated, the presence of nonsolvent does not always lead to phase separation. Therefore, the measurement of the change in the composition of the polymer solution that dictates the phase separation during drying is of prime importance.

### Correlating the morphology of the polymer film and the composition path

The morphology of the final polymer film obtained by EIPS technique can be tailored by the choice of the polymer, by altering the concentration of nonsolvent and polymer in the casting solution, initial thickness of the casting solution, relative humidity and temperature. Although, there are theoretical studies on the morphology of the polymer films prepared by EIPS technique,^26–28^ to the best of our knowledge, there are no experimental studies that comprehend the effect of different parameters on the evolution of composition of the casting solution and the resulting final film morphology.

### Morphology of the polymer films

**Fig. 3:**
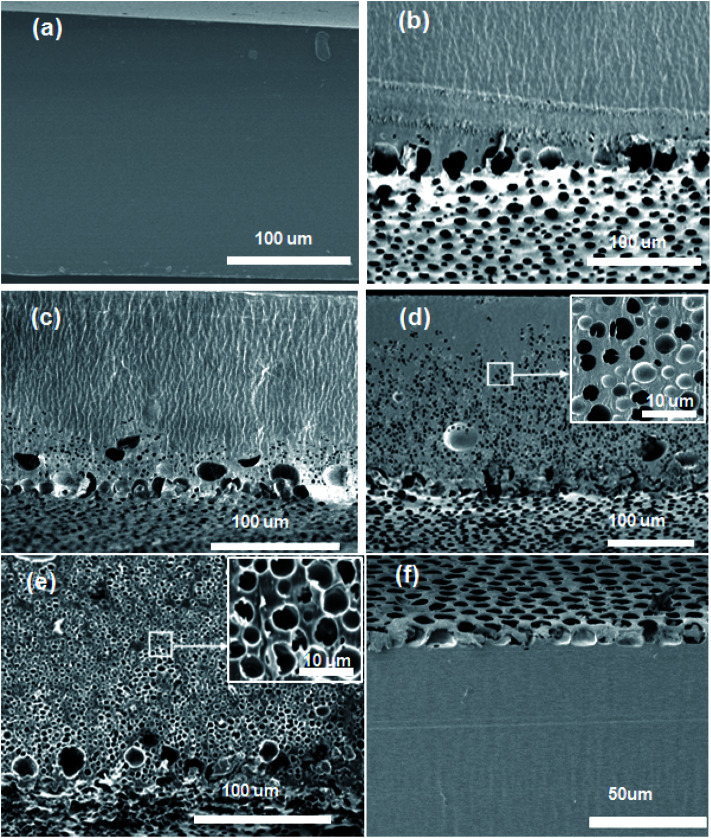
The cross-sectional view imaged by scanning electron microscopy show the different morphology of the PMMA polymer films fabricated *via* the evaporation induced phase separation technique (EIPS) technique: (a) non porous polymer film (b) polymer film with porous bottom surface (c) asymmetric porous film with a thick dense skin layer at the top (d) asymmetric porous film with a thinner dense skin layer at the top (e) symmetric porous film where pores are distributed across the entire film (f) film with porous top surface. The insets in (d) and (e) show zoomed view of film cross section.

Table 1 summarizes the composition of PMMA, THF and water in the homogeneous casting solution and the experimental conditions used for the fabrication of the polymer films. The polymer films formed under various experimental conditions were transparent, semi-transparent or opaque. For example, the films formed by casting solutions labeled S_1W5P_, S_2W5PT_ and S_3W5PT_ were transparent. The polymer films formed when casting solutions represented by S_2W5P_, S_4W10P_, S_4W15P_, S_3W5PTh_, S_4W5PT_, S_0W5PH_ and S_1W5PH_ were dried, a semitransparent film was obtained. However, drying of samples S_3W5P_ and S_4W5P_ resulted in polymer films that were opaque. The microstructures of the cross section of all the polymer films fabricated visualized by scanning electron microscopy (SEM) revealed remarkably different features, which can be classified into five classes as represented by the microstructures shown in Fig. 3. The SEM images for the surface of the film in contact with the glass substrate are shown in the ESI Fig. SI 5.† It must be noted that, the cross-sections show larger or distorted pores, an artifact of the use of microtome for the cutting of polymer films for the electron microscopy observations. The microstructures in Fig. 3 correspond to the polymer films formed by the drying of casting solutions S_1W5P_ (nonporous), S_2W5P_ (bottom surface pore), S_4W10P_ and S_3W5P_ (asymmetric pore), S_4W5P_ (symmetric pore) and S_4W5PT_ (top surface pore). The non-porous structure similar to that shown in Fig. 3(a) was obtained from the casting solution corresponding to S_1W5P_, S_2W5PT_ (Fig. SI 9a, in ESI†) and S_3W5PT_ (Fig. SI 9b, in ESI†). The spherical uniform pores at the location of the casting solution that was contact with the substrate as shown in Fig. 3(b) was obtained in the films fabricated using S_2W5P_ and S_4W15P_ (Fig. SI 6, in ESI†). Asymmetric porous film with 89.4%, 66.9% and 29% thick dense skin layer were obtained from S_3W5PTh_ (Fig. SI 7, in ESI†), S_4W10P_ (Fig. 3(c)) and S_3W5P_ (Fig. 3(d)) samples respectively. Symmetric porous film was obtained from S_4W5P_ sample where pores were distributed throughout the film as shown in Fig. 3(e). Finally, films with pores only at the top surface pore was obtained by drying samples S_4W5PT_ (Fig. 3(e)), S_0W5PH_ (Fig. SI 8a, in ESI†) and S_1W5PH_ (Fig. SI 8b, in ESI†).

**Fig. 4:**
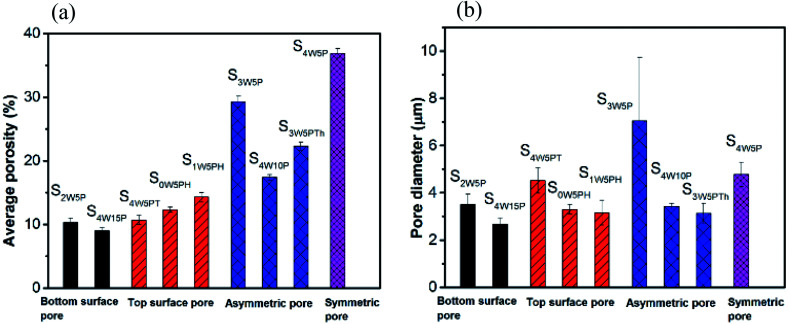
Average porosity of the film (a) and average pore diameter (b) of the film surface in contact with the glass substrate obtained under different experimental conditions.

The average porosity of the polymer films and the diameter of the pores at the film surface in contact with glass substrate were observed to depend on initial composition as well as experimental conditions as shown in Fig. 4. The effect of water concentration (1–4 wt%) on the morphology of the polymer film was studied by considering S_1W5P_, S_2W5P_, S_3W5P_ and S_4W5P_ where all other variables such as temperature (30 °C), relative humidity (60%), casting solution thickness (5 mm) and PMMA concentration (5 wt%) were kept constant. At 1 wt% water, non-porous film was formed while for 2, 3 and 4 wt% water porous films with asymmetric to symmetric pore distribution were formed (Table 1 and Fig. 3). In spite of presence of water (non-solvent), S_1W5P_ forms nonporous film and with increasing water concentration, the porosity increases from 10.3% for S_2W5P_ to 36.9% for S_4W5P_ sample as shown in Fig. 4 and tabulated in Table 1. Similarly, keeping water concentration at 4 wt% and increasing the PMMA concentration (5 to 10 and 15 wt%) in the casting solution the porosity decreased from 36.9% for S_4W5P_ to 17.4 for S_4W10P_ and 9% for S_4W15P_ as shown in Fig. 4. The thickness of the dry polymer film left behind post evaporation depends on the thickness of the casting solution. For example, thickness of the dry polymer films obtained from 5 mm and 1 mm thick casting solutions were approximately 150 μm (S_3W5P_) and 28 μm (S_3W5PTh_) respectively. As evident in Table 1, while a 5 mm thick casting solution leads to asymmetric polymer film with 29.3% porosity (S_3W5P_), a decrease in initial casting solution thickness from 5 to 1 mm *i.e.*, sample S_3W5PTh_ leaves an asymmetric film with a lower porosity of 22.3%, all other parameters being kept constant. In general, as can be inferred from Tables 1 and 2, the porosity of the polymer films were observed to increase with increase in the ratio of water/PMMA at the point of phase separation. On the other hand porosity was observed to decrease with increase in temperature from 30 °C to 50 °C, as observed in sample S_4W5P_ (overall porosity 36.9%) and S_4W5PT_ (overall porosity 10.7%). The increase in relative humidity (74% RH) shows a transition from non-porous film (for S_1W5P_ at 60% RH) to porous film as observed in sample S_1W5PH_ in Fig. 3. Interestingly, the casting solution without water at higher RH (74%) shows porous film under similar experimental condition of S_1W5P_. In general, with increasing water (non solvent) concentration pore size should increase as reported in literature.^3^ However, in this study, as the pores were distributed asymmetrically in the substrate–surface interface and across the film thickness the pore size does not directly proportional to the water concentration rather depends on the different variables in the casting solution and casting conditions. Moreover, it was observed that the diameter of the pores at the substrate–surface interface was larger than pores across the cross-section of the films. The data presented in Fig. 4 reveals that by careful choice of initial composition and casting conditions the pore morphology can be tuned.

**Table 2:** Phase separation time and water/PMMA ratio at instant of phase separation for the various casting solution used for the polymer film fabrication under different experimental conditions

Sample	Phase separation time (min)	Water/PMMA ratio at the moment of phase separation	Porous layer thickness (%)
S_1W5P_	—	—	—
S_2W5P_	60 ± 2	1.2	Bottom layer pore
S_3W5P_	55 ± 1	1.5	71
S_4W5P_	51 ± 1	1.6	100
S_4W10P_	40 ± 1	0.6	33.9
S_4W15P_	29 ± 2	0.4	Bottom layer pore
S_3W5PTh_	16 ± 1	1.1	12.18
S_2W5PT_	—	—	—
S_3W5PT_	—	—	—
S_4W5PT_	35 ± 1	—	Top layer pore
S_0W5PH_	69 ± 1	—	Top layer pore
S_1W5PH_	63 ± 1	0.8	Top layer pore

The dry PMMA films, both non porous and porous, were also characterized by FTIR and XRD. The characteristic features of spectra (Fig. SI 10 and SI 11 shown in ESI†) were similar to reported literature^29–32^ and there were no noticeable difference for different types of porous films.

To understand these observations and the distribution of pores across the film thickness, the determination of composition of the casting solution during evaporation in relation to the phase diagram is necessary. From the average value of the compositions of the casting solution one can understand the feasibility of pore formation. But for asymmetric and symmetric pore distribution as in case of S_2W5P_, S_3W5P_ and S_4W5P_, the measurement of average composition is not sufficient. Therefore the CP for both top (air–solution interface) and bottom (solution–substrate interface) surface of the polymer solution during drying were measured.

### Evolution of the composition (composition path) of the PMMA–THF–H_2_O solution during film formation

**Fig. 5:**
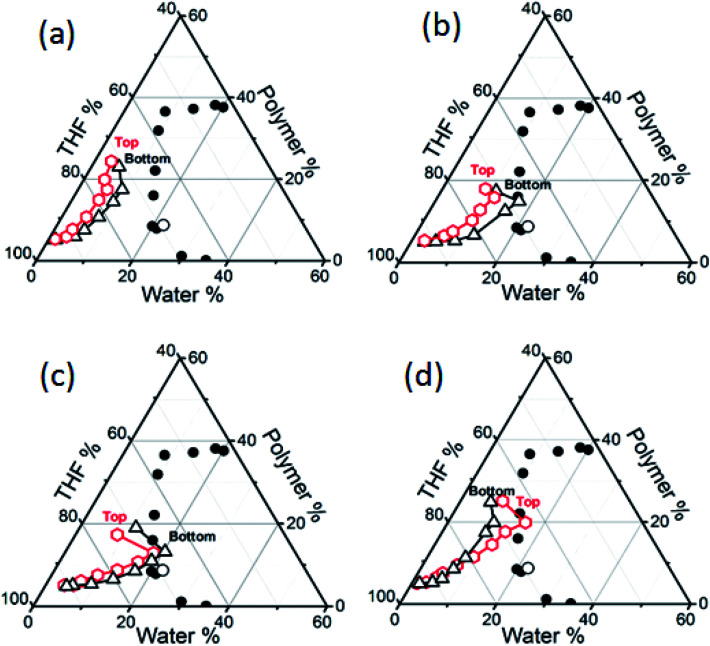
The evolution of composition of (a) S_1W5P_, (b) S_2W5P_, (c) S_4W5P_ and (d) S_1W5PH_ casting solutions during the fabrication of PMMA films by EIPS. The open hexagonal and triangular symbols represent the composition measured experimentally by conductivity for top and bottom surface of the casting solution respectively. The line connecting the composition change during drying are the composition paths (CPs). For S_1W5P_ sample, the CP remains in the single phase region during the course of drying resulting nonporous film. On the other hand, for S_2W5P_ the CP measured at the bottom surface crosses the binodal curve but that corresponding to the top surface does not touch the phase boundary. This leads to asymmetric porous films with dense top layer. While for S_4W5P_, the CPs at both top and bottom surfaces crosses the binodal curve and therefore a symmetric porous film is formed. For S_1W5PH_, only the CP pertaining to the top surface crosses the phase boundary.

During the course of polymer film formation, the mass of the polymer in the casting solution remains constant, however, the concentration of the polymer in the solution increases as both THF and water evaporate. Since PMMA and THF in the three component casting solution are non conductive and the ionic conductivity of PMMA–THF–water mixture depends on the concentration of water in the mixture, the conductivity of the casting solution increases with time since the THF in the mixture evaporates at a faster rate. In our experiments, the change in the composition of the casting solution, *i.e.*, the composition path (CP) during the formation of the polymer film was measured for (i) the top surface – that is – in the vicinity of the solution–air interface and (ii) the bottom surface – that is – in the vicinity of the solution–substrate interface. The composition path (CP) obtained by the conductivity measurements were plotted on the ternary phase diagram of the PMMA–THF–H_2_O system that was determined by cloud points measurement (Fig. 5). In general, the conductivity of the solution was observed to increase during initial stages of drying and after certain time, a decrease was observed for all the solutions considered in this study. Using visual observation, turbidity measurements (Fig. SI 3, in ESI†) and optical microscopy, it was confirmed that the decrease in the conductivity was due to the formation of water drops due to liquid–liquid phase separation. Since the majority of water in the casting solution forms the disperse phase, the conductivity decreases drastically.

As shown in Fig. 5, the measurement of CP for the PMMA–THF–H_2_O casting solutions under different conditions shows that the composition of the casting solution at the top surface (solution–air interface) was different than that at the bottom surface (solution–substrate interface). This implies that during the film formation, a concentration gradient develops across the casting solution thickness. Fig. 5(a)–(d) show the CPs for the PMMA–THF–H_2_O casting solutions represented by S_1W5P_, S_2W5P_, S_4W5P_ and S_1W5PH_. The CP measured during the evaporation of solvent and non-solvent was significantly different and could be classified into four different categories depending on the morphology of the polymer film. In some cases, the CP for both the top surface and bottom surface remain in the single-phase region throughout the drying period as shown for the casting solution labeled S_1W5P_ (case I). Therefore liquid–liquid phase separation does not arise and drying of S_1W5P_ forms a nonporous or dense film. When the casting solution labeled S_2W5P_ was dried, while the bottom surface CP crosses the phase boundary, the CP for the top surface remains in the single phase region (case II). Therefore liquid–liquid phase separation occurs at the bottom surface leading to porous structure at the bottom surface with nonporous top layers as shown in Fig. 3(b). Another class of behavior, where the CPs for both the top and bottom surfaces crosses the phase boundary (case III) was observed for the drying of casting solution labeled S_4W5P_. Therefore phase separation occurs across all the layers resulting in pores across the entire film, *i.e.*, symmetric porous film. Finally, in certain cases, only the CP for the top surface crosses the phase boundary and the CP for the bottom surface remains in the single phase region (case IV), which was observed during the drying of casting solution S_1W5PH_. This results in films with porous top layer with nonporous bottom layer.

The evolution of the CP was measured by considering casting solutions of varying polymer and water concentration and at different casting conditions. For example, the asymmetric porous film where pores were present at the bottom surface as well as across some cross section as in case of S_3W5P_, S_4W10P_ and S_3W5PTh_ were observed to exhibit a CP similar to case II, *i.e.*, CP for the top surface remain in the single phase region and the CP for the bottom surface crosses the phase boundary (Fig. SI 12, in ESI†). However, it must be noted that the composition corresponding to cross-over occurs at different water/PMMA ratio and a higher water/PMMA ratio leads to pores over larger thickness of the polymer film (Table 2). As for the samples S_2W5P_, S_3W5P_ and S_4W5P_, at constant 5 wt% initial PMMA concentration, the water/PMMA ratio at the moment of phase separation is 1.2, 1.5 and 1.6 respectively. In spite of similar trend in the variation of CP for S_2W5P_ and S_3W5P_, the films fabricated from S_3W5P_ had a larger porous layer than S_2W5P_ and films prepared from S_4W5P_ had pores across the entire film thickness. It was also observed that S_4W5P_ casting solution phase separates at earlier time than S_3W5P_ and S_2W5P_ as was discussed in Section 3.2 and from the data shown in Table 2. On the other hand, for S_4W10P_ and S_4W15P_ samples, the initial PMMA concentration was as high as 10 and 15 wt% respectively. This leads to a higher concentration of PMMA in the casting solution at the time of phase separation and the water/PMMA ratio was very low – 0.6 and 0.4 respectively *i.e.*, PMMA/water ratio increased from 1.67 to 2. Consequently the PMMA film corresponding to S_4W10P_ showed more porosity (less dense layer thickness) and the pores were distributed at the bottom surface as well as across the film thickness while in the polymer film fabricated from S_4W15P_, the pores were distributed only at the bottom surface (more dense layer thickness). Similar observation that an increasing polymer to water ratio leads to a film increased dense skin layer thickness was reported by Altinkaya *et al.*^22^ A decrease in the initial casting solution thickness from 5 mm (sample S_3W5P_) to 1 mm (sample S_3W5PTh_) showed a decrease in water/PMMA ratio from 1.5 to 1.1 at the point of phase separation (Table 2). This results decrease in the thickness of the porous layer and the formation of pores only at the bottom surface (hence less porosity) for the PMMA films obtained from S_3W5PTh_. Matsuyama *et al.*^23^ reported similar observations when the casting solution thickness was decreased from 254 μm to 127 μm. On the other hand, Altinkaya *et al.*^22^ showed the formation of polymer films of same porosity when casting solution thickness was reduced from 300 μm to 200 μm. At higher temperature (50 °C), the CP for S_2W5PT_ and S_3W5PT_ casting solution are expected to be similar to case-I and the CP for the casting solution S_4W5PT_ was similar to case-IV. Although the CP at 50 °C was not measured, the SEM images show non-porous structures for S_2W5PT_ and S_3W5PT_ samples and only top surface pores for S_4W5PT_ sample. We speculate that the CP for both the surface did not cross the phase boundary since THF and water evaporate at faster rate at 50 °C. However, due to higher initial water concentration (4 wt%) in the casting solution S_4W5PT_, in spite of increase in the rate of evaporation of both THF and water, we postulate a higher concentration of water at the top surface compared to the bottom surface and the phase separation to occur at only the top surface. As a result, the composition of the top surface intersects the phase boundary. Young *et al.*^33^ reported that a rise in the evaporation temperature during the dry-cast process changed the membrane structure from a particulate to dense film morphology, in line with results reported in this work. For the case of S_0W5PH_ and S_1W5PH_ relative humidity during the film formation was maintained at 74%. The CP for these samples were similar to case-IV *i.e.*, the CP for the top surface crosses the phase boundary and the CP for the bottom surface remains in the single phase region. The evaporation of the solvent and the nonsolvent from the air–solution interface lead to the evaporative cooling effect. While the initial temperature of the solution was 30.1 °C, the temperature was observed to drop to 25 °C after 5 minutes. Therefore, at higher relative humidity, the water drops nucleate at the top surface (air–solution interface) ultimately leading to a polymer film with only top layer pores. The evaporative cooling effect was also observed by Shojaie *et al.*^24^ and Altinkaya *et al.*^22^ for polymer films fabricated from EIPS for cellulose acetate–acetone–water system. Prior to this study, the CPs obtained theoretically was extrapolated up to the two phase region to explain the pore morphology in the polymer films obtained experimentally.^6,19,26^ The results discussed in this section demonstrate an understanding of the ultimate morphology of the polymer films under different experimental conditions. The distribution of pores in the final polymeric films obtained by evaporation induced phase separation depends on the evolution of the composition of the mixture during drying and the phase behavior of the constituent mixture.

### Effect of molecular weight (*M*_w_) and polydispersity index (PDI) on the morphology of polymer films

**Fig. 6:**
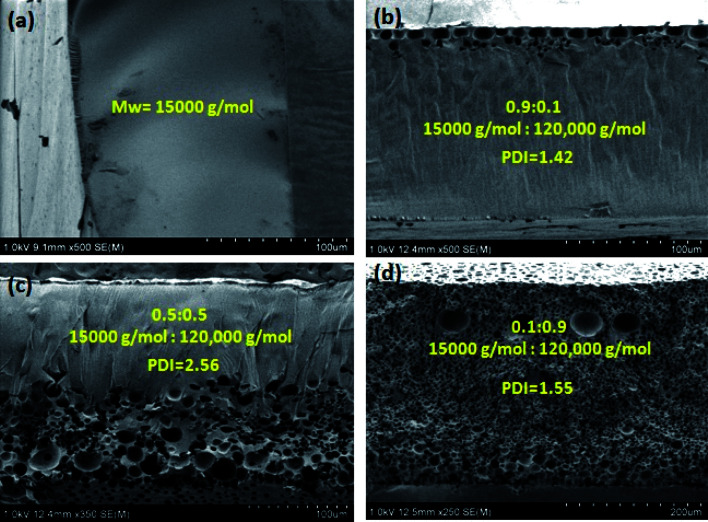
The cross-sectional view of PMMA films of different morphology fabricated *via* the evaporation induced phase separation technique (EIPS) by evaporating PMMA–THF–H_2_O mixture containing (i) low molecular weight PMMA (*M*_w_ = 15 000 g mol^−1^) and (ii) a mixture of low (*M*_w_ = 15 000 g mol^−1^) and high molecular weight (*M*_w_ = 120 000 g mol^−1^) PMMA. The composition of the casting solution and experimental conditions are similar to S_4W5P_. (a) Low molecular weight PMMA (*M*_w_ = 15 000 g mol^−1^) and PDI = 1. (b), (c) and (d) are from a mixture of low *M*_w_ (15 000 g mol^−1^) and higher *M*_w_ (120 000 g mol^−1^) PMMA in the ratio of 0.9 : 0.1; 0.5 : 0.5 and 0.1 : 0.9, respectively.

The PMMA films were fabricated by evaporating PMMA–THF–H_2_O, however, with solutions containing (i) low molecular weight PMMA of *M*_w_ = 15 000 g mol^−1^ and (ii) a mixture of low (*M*_w_ = 15 000 g mol^−1^) and high molecular weight (*M*_w_ = 120 000 g mol^−1^) PMMA. The mass ratio of low and high molecular weight PMMA in the casting solution was changed such that the polydispersity index, PDI of PMMA in the casting solution varied from 1 to 2.56. The composition of the casting solution and the experimental conditions were similar to those used during the evaporation of sample labeled S_4W5P_ (*i.e.*, the casting solution that form porous film shown in Fig. 3(e)). The SEM images of the polymer film shown in Fig. 6 obtained after the complete evaporation of THF and H_2_O in the casting solution elucidate the effect of *M*_w_ and PDI of PMMA on the morphology of the final polymer film. The microstructures shown in Fig. 6 correspond to the cross section of the films formed by drying casting solution containing (a) 15 000 g mol^−1^ molecular weight PMMA, PDI = 1.0 (b) mixture of 15 000 g mol^−1^ molecular weight and 120 000 g mol^−1^ molecular weight PMMA (0.9 : 0.1 weight ratio), PDI = 1.42 (c) mixture of 15 000 g mol^−1^ molecular weight and 120 000 g mol^−1^ molecular weight PMMA (0.5 : 0.5 weight ratio), PDI = 2.56 (d) mixture of 15 000 g mol^−1^ molecular weight and 120 000 g mol^−1^ molecular weight PMMA (0.1 : 0.9 weight ratio), PDI = 1.55.

As evident from Fig. 6(a), a nonporous film was obtained when the casting solution containing low molecular weight PMMA was dried. Moreover, the PMMA film formed was transparent and not stable (*i.e.*, the film was not a free standing), most likely due to inferior mechanical strength of the low *M*_w_ PMMA. Since the low *M*_w_ PMMA is more soluble than higher *M*_w_ PMMA, it appears that S_4W5P_ (5 wt% PMMA–4 wt% water) sample containing low *M*_w_ PMMA does not cross the phase boundary during film formation. As a result, the phase separation does not occur resulting a nonporous film. However, the addition of a small quantity of high *M*_w_ PMMA (10 wt%) lead to a PMMA film with pores only at the top surface as shown in Fig. 6(b). With further increase in the concentration of high *M*_w_ PMMA (to 50 wt%), the final polymer film was found contain pores across half of the cross section and the other half was found to be non-porous as evident in Fig. 6(c). With the high *M*_w_ PMMA fraction in the casting solution increased to 90%, the pores appear throughout the cross-section of the film as shown in Fig. 6(d). Therefore, the morphology of the polymer film can be tuned just by varying the composition of low and high *M*_w_ PMMA in the casting solution.

## Summary and conclusions

A systematic experimental investigation of the formation of polymer films with controlled porous structure *via* evaporation induced phase separation is carried out. The emphasis of the work is on the analysis of the evolution of the microstructure during the film formation and to correlate the morphology of the polymer film with the change in the composition of the casting solution at the top surface and the bottom surface. The state of the polymer solution during evaporation is comprehensively analyzed by optical microscopy, digital imaging, turbidity measurements and the composition path is determined by monitoring the temporal evolution of the conductivity of the solution. We show that the porous structures are formed by nucleation and growth of nonsolvent droplets through liquid–liquid phase separation. To obtain porous films from a polymer–solvent–nonsolvent system, the liquid–liquid phase separation is necessary, *i.e.*, the CP during the drying process must intersect the cloud-point (binodal) curve. As discussed, for a constant polymer concentration in the three component mixture, the ratio of concentration of non solvent to polymer in the casting solution at the point of phase separation determines the distribution of pores across the film thickness. Therefore, the measurement of CP for both top and bottom surface of the casting solution for a given polymer–solvent–nonsolvent system can be used to directly infer the final film morphology. However, the CP depends on several factors such as initial composition of the casting solution as well as casting conditions such as solution thickness, relative humidity and temperature. Therefore, a detailed knowledge of the composition path in relation to phase behavior is of prime importance to design and engineer polymer films of required morphology for a given application. Another important outcome of this study is that, the morphology of the PMMA film can be tuned just by varying the composition of low and high *M*_w_ PMMA in the casting solution. Considering enormous interest in the fabrication of polymer films with tailored pore structure starting from several polymer–solvent–nonsolvent systems, this study is expected to help in the fundamental understanding of methodology to achieve this objective.

## Conflicts of interest

There are no conflicts to declare.

## Supplementary Material

RA-009-C9RA01331H-s001
