# Supplementary material for: Tailoring pore distribution in polymer films via evaporation induced phase separation
Source: RSC Adv. 2019 May 17;9(27):15593–605. doi: 10.1039/c9ra01331h (PMC9064270; doi:10.1039/c9ra01331h)
Supplement: RA-009-C9RA01331H-s001 [file RA-009-C9RA01331H-s001.pdf]

Electronic Supplementary Material (ESI) for RSC Advances.
This journal is © The Royal Society of Chemistry 2019

Supplementary Information

Tailoring pore distribution in polymer films via evaporation induced phase separation

Rumiaya Pervin [1,2], Pijush Ghosh [2],Madivala G. Basavaraj[1] *

[1]Department of Chemical Engineering, Indian Institute of Technology Madras, Chennai, 600036, India

[2] Department of Applied Mechanics, Indian Institute of Technology Madras, Chennai, 600036, India

*Corresponding author: basa@iitm.ac.in

Measurement of cloud point and critical point for PMMA-THF-H_2O ternary system

The cloud points were obtained by the addition of water to a clear (transparent) PMMA-THF solution (Figure S1) of various different compositions. PMMA dissolves in THF in all composition ranges, but insoluble in water. However, water mixes (miscible) with THF over the entire composition range at 30°C. With addition of small amount of water to the homogeneous PMMA-THF solution, the solution remained homogeneous and single phase. At certain water concentration, as shown in Figure SI 1b, the clear homogeneous solution turned turbid as the solution intersected the binodal curve in the phase diagram of the ternary mixture. The appearance of cloudiness that sustained was noted as cloud point and eventually the solution phase separated into two distinct layers (a clear polymer rich and a clear polymer lean phase) in thermodynamic equilibrium in about 5 hours as shown in Figure SI 1. When these two distinct equilibrium phases (shown in Figure SI 1e) were observed under an optical microscope, there were no droplets in each of the phases. A water soluble fluorescent die added to the mixture

confirmed the presence of water in both the phases. Therefore, both PMMA rich and PMMA lean phase contain THF as well as water. The critical point was obtained by measuring the volume of the polymer rich and polymer lean phases in the mixture at equilibrium. At the critical point, the ratio of these two volumes is unity. The concentration of polymer at that point is called as critical concentration. The composition of the three component mixtures at the critical point at 30°C was – 8.6, 69.1 and 22.3 wt % of PMMA, THF and water respectively. Above 40 % polymer concentration, it was not feasible to measure the cloud points experimentally as the solutions were highly viscous.

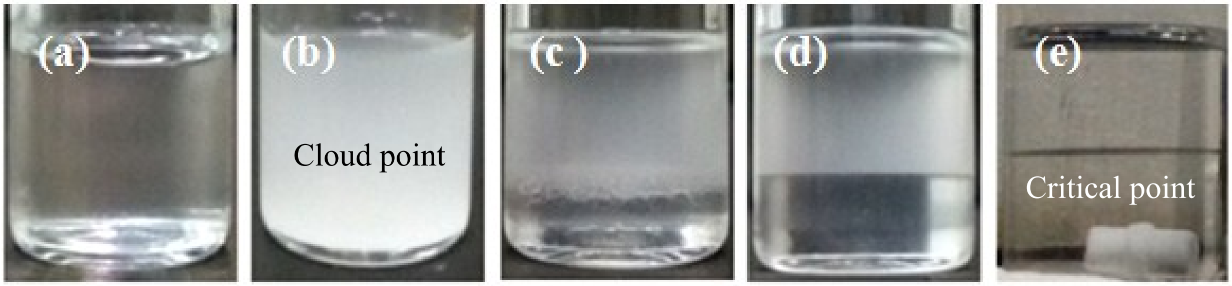

Figure SI 1. Demonstration of phase separation in the ternary PMMA-THF-water mixture: (a) PMMA-THF clear solution containing 1g PMMA and 8g THF, (b) Turbid solution after the drop wise addition of approximately 2.6 g water to the PMMA-THF homogeneous mixture resulting in a ternary PMMA-THF-Water of 8.6-69.1-22.3 composition. This composition at which the turbidity just appears in the mixture due to the formation of water droplets is known as the cloud point. At this point two phases namely, polymer rich and polymer lean phase form. In the images (c) and (d) respectively at 26 min and 60 min after the nucleation of water drops, the density difference between the two phases and coalescence of drops lead to the formation of clear bottom phase. (e) Polymer rich and polymer lean phases attain equilibrium in about 5 hrs and form two clear distinct equilibrium phases.

Microstructure of the ternary PMMA-THF-H_2O solution

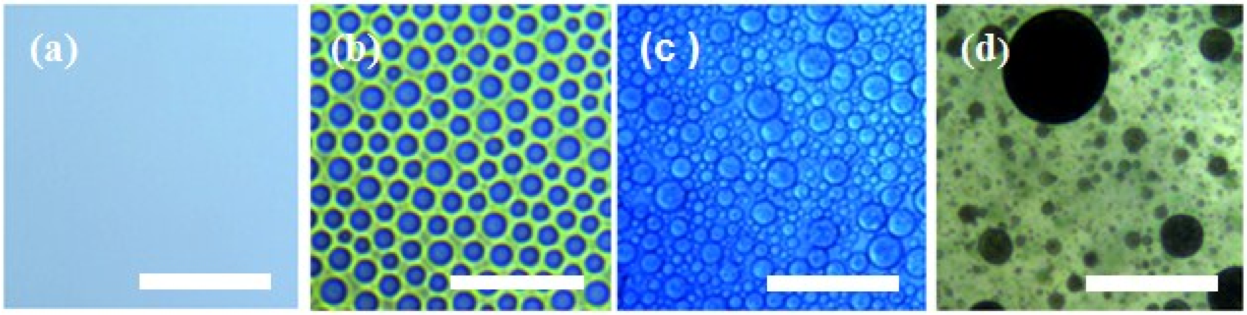

Figure SI 2. The microstructure of the bulk solution with different composition of PMMA-THF-water: (a) at 5-91-4 composition, the mixture was in single phase region and was a clear homogeneous solution, (b) at 10-70-20 composition near the critical point, a homogeneous distribution of water droplets in the two phase solution was observed, (c) at composition such as 5-65-30, far from the critical point, there were polydisperse water droplets in the two phase mixture and (d) in mixtures with higher water concentration such as 10-50-40, the polymer precipitation was observed. Scale bar in each image is 5 μm.

To analyze the microstructure of each solution prepared to generate the phase diagram, brightfield optical microscopy was used. To this end, several PMMA-THF-water solutions of different position corresponding to different regions in the phase diagram were prepared and observed under an optical microscope. In the single phase region, the mixture of three components (PMMA-THF-water) was homogeneous and clear irrespective of the composition. For instance, the solution with composition 5-91-4 of PMMA-THF-water was a clear solution as shown in Figure SI 2a. Whereas, the solution that turned turbid after the addition of water clearly showed the presence of water droplets. The composition near to critical point showed uniform distribution of water droplets which were stable against coalescence or sedimentation, as in the microstructure corresponding to a composition of 10-70-20 of PMMA-THF-water shown in

Figure SI 2b. Far from the critical point, the mixture inhomogeneous and contained water droplets that grew in size due to coalescence as was observed for the composition 5-65-30 of PMMA-THF-H_2O in Figure SI 2c. In addition, it was observed that for 10-50-40 of PMMA-THF-H_2O, i.e., for higher water concentration, the phase separation was accompanied by the precipitation of the polymer as can be seen from Figure SI 2d. It is clear that phase diagram can be used to identify morphology of the PMMA-THF-H_2O solution of a given composition. However, in order to understand the relationship between the phase behavior of the solution and the morphology of the polymer film, the measurement of the change in composition of the casting solution during film formation is necessary. Therefore the change in composition of PMMA-THF-H_2O solution during film formation was monitored.

Measurement of turbidity during film formation and on set of phase separation

From visual observation and microscopy analysis, it was observed that phase separation occurs at a faster rate when the concentration of polymer or water in the mixture was increased. This was also evident from the rapid changes in the turbidity of the casting solution. After the phase separation, the casting solution rapidly changed into a solid film within 5 to 7 minutes.

The change in the turbidity of the PMMA-THF-H_2O casting solutions was quantified by measuring the intensity of transmitted light. As shown in Figure SI 3, the intensity of transmitted light (ITL) for S_{2W5P}, S_{3W5P} and S_{4W5P} was constant initially and starts to decrease sharply. The time at which intensity decreased sharply was observed to coincide with visual change in the appearance of the casting solution from clear to turbid. From Figure SI 3, this sharp decrease was observed at 51, 55 and 60th minute of drying for the casting solutions S_{4W5P}, S_{3W5P} and S_{2W5P} respectively. Interestingly this coincided with the nucleation of water droplets observed in time

resolved microscopy images. With time, the ITL decreased sharply and became constant during final stages of film formation when the casting solution appeared visually opaque. On the contrary, during the drying of S_{1W5P}, ITL was constant throughout the course of drying confirming the absence of any liquid-liquid phase separation (and hence water droplet formation) consistent with the microscopy observation.

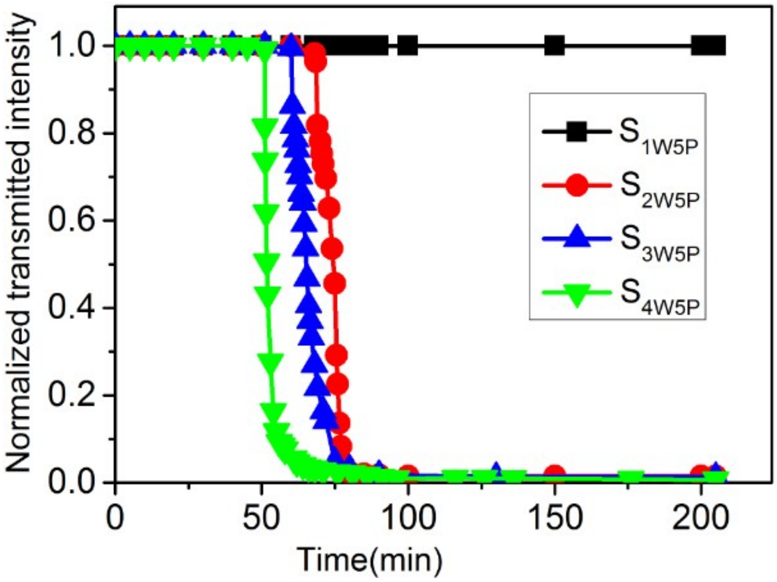

Figure SI 3. Measurement of turbidity of S_{1W5P}, S_{2W5P}, S_{3W5P} and S_{4W5P} samples during the film formation by light transmittance. Sharp fall in the normalized intensity is due to change in turbidity as a result of the nucleation of water droplets at 51, 55 and 60th min respectively for drying of S_{4W5P}, S_{3W5P} and S_{2W5P}. In S_{1W5P}, there is no change in turbidity as water droplets do not nucleate.

Conductivity calibration for THF-water mixture

The conductivity measurement was used to construct the composition path. The permittivity or dielectric constant of water and THF are 80.4 and 7.6 respectively. Therefore THF solely is not conductive and the conductivity of water depends on the presence of dissolved ions. THF evaporates faster rate than water. Hence, the evaporation of THF lead to an increase in the concentration of water and that of ions in the casting solution and correspondingly an increase in the conductivity of the casting solution was observed. In the conductivity experiments, a CM-

230 conductivity monitor that can measure conductivity in 0.1-2000μS/cm range at 0.1μS/cm resolution was used. The original probe of the meter being larger in length was not suited to measure the conductivity of the solution of smaller volume. Therefore this probe was replaced with Teflon coated alluminium rod of 1mm diameter constructed in-house. Only the tip of the probe (the circular end) i.e., the portion not coated with Teflon was available for the conductivity measurement. This conductivity meter was capable of measuring the conductivity of a solution at any depth. The cell constant, determined based on the conductivity of 0.84 mM aqueous NaCl solution (100 μS/cm) and other standard solution (1413 μS/cm) supplied by HAANA was 1.4 cm^{-1}. The conductivity was measured for THF, water and the mixture of THF-water (with different concentrations) at 25ºC and 30°C. A known quantity of THF and water was taken in a 5 mm diameter glass vial to prepare THF-water mixture of different concentrations. The probe was then inserted into the solution and the conductivity was recorded. With increasing the concentration of water, the conductivity was observed to increase as expected. A calibration curve was obtained by plotting conductivity as a function of the mole percent of water in the mixture (Figure SI 4). The weight of the THF and water was measured by an analytical balance with accuracy up to four decimal places.

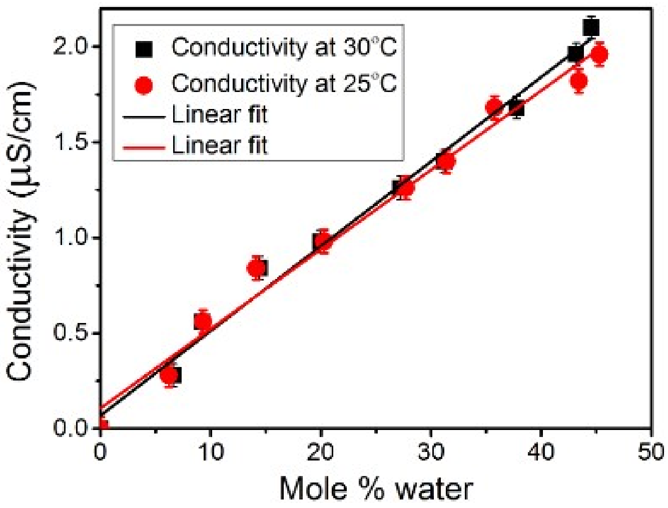

Figure SI 4. The conductivity of THF- water mixture as a function of water concentration (mole %). At zero water concentration the conductivity value is zero and with increasing water concentration conductivity value increases.

Table SIT 1. Calulation of composition path (CP) from conductivity value for S_{4W5P} sample for the top surface. CP for the bottom surface was measured similar way by measuring conductivity at the bottom surface.

The data shown in Table SIT 1 corresponds to the composition path shown by red hexagonal symbols in Figure 5 c) in the manuscript.

Conducta -nce (µS)	Conduct -ivity (µS/cm)	Solution weight (gm)	Polymer weight (gm)	Poly- mer %	Water +THF(gm)	Water (%) from calibrati- on	Water in solution (gm)	THF in solution (gm)	Water (%)	THF (%)
0.7	0.98	6.45	0.3225	5.0	6.1275	5.734	0.3513	5.7761	5.45	89.55
0.6	0.84	6.34	0.3225	5.08	6.0175	5.087	0.3061	5.7114	4.83	90.08
0.5	0.7	6.137	0.3225	5.25	5.8145	3.492	0.2030	5.6114	3.31	91.44
0.6	0.84	5.853	0.3225	5.51	5.5305	4.723	0.2612	5.2693	4.46	90.03
0.7	0.98	5.775	0.3225	5.58	5.4525	5.987	0.3264	5.1260	5.65	88.76
0.8	1.12	5.298	0.3225	6.08	4.9755	7.243	0.3604	4.6151	6.80	87.11
0.9	1.26	5.028	0.3225	6.41	4.7055	8.694	0.4091	4.2964	8.14	85.45
1	1.4	4.219	0.3225	7.64	3.8965	10.135	0.3949	3.5016	9.36	82.99
1.2	1.68	3.864	0.3225	8.35	3.5415	13.762	0.4874	3.0541	12.61	79.04
1.4	1.96	3.509	0.3225	9.19	3.1865	16.165	0.5151	2.6714	14.68	76.13
1.7	2.38	2.982	0.3225	10.81	2.6595	18.124	0.4820	2.1774	16.16	73.02
2	2.8	2.722	0.3225	11.85	2.3995	20.535	0.4927	1.9067	18.10	70.05
1	1.4	2.294	0.3225	19.47	1.9715	9.653	0.1903	1.7812	8.29	71.13

Morphology of PMMA films

Effect of water concentration

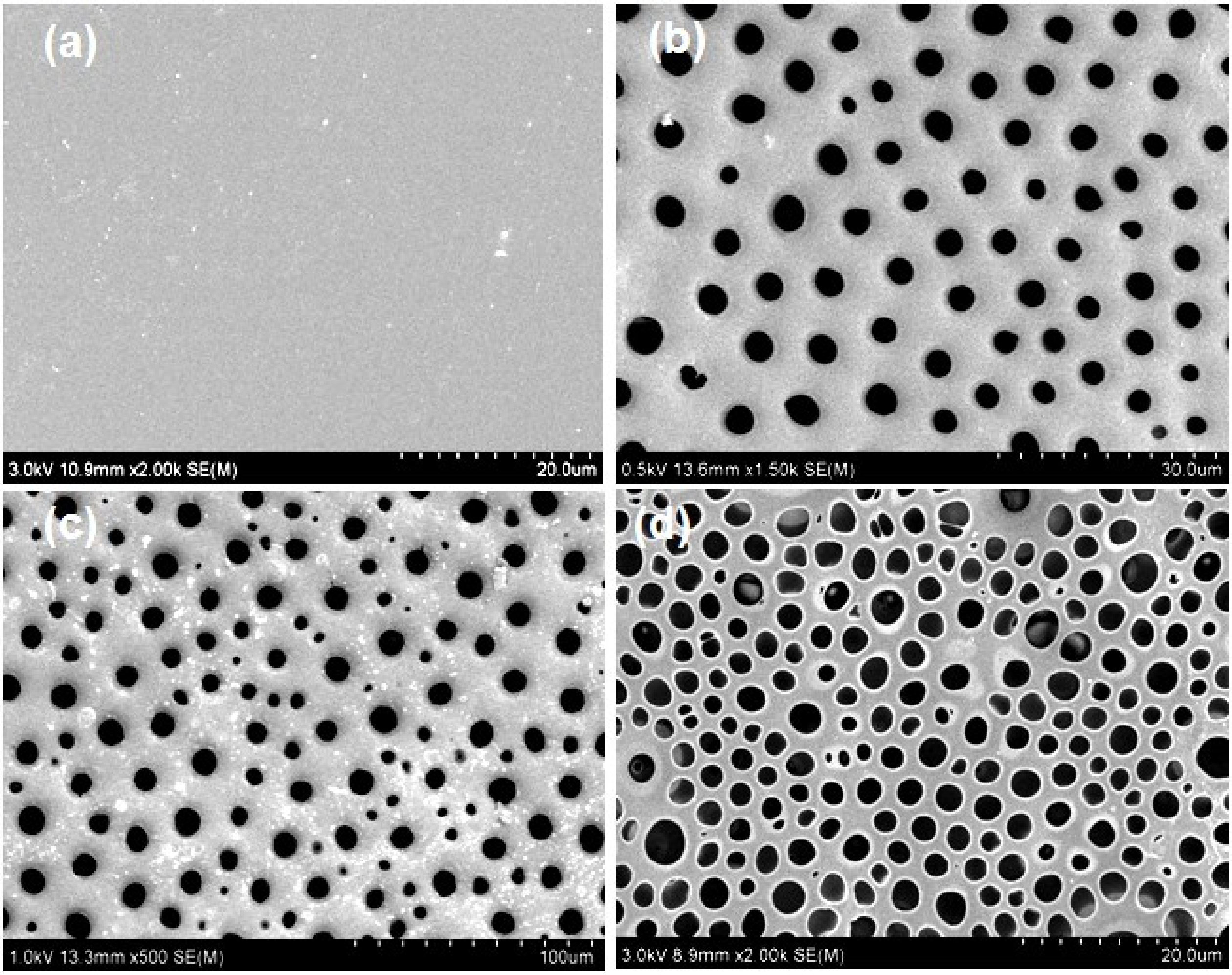

Figure SI 5. The SEM micrographs of the PMMA film surface in contact with the surface of the Petri dish fabricated by casting (a) S_{1W5P}, (b) S_{2W5P}, (c) S_{3W5P} and (d) S_{4W5P} samples respectively where water concentration varies from 1 to 4 wt %. The PMMA concentration was 5 wt% for all the cases. A non-porous structure was obtained for 1 wt % water and porous structures were formed for 2, 3 and 4 wt% water.

Effect of PMMA concentration

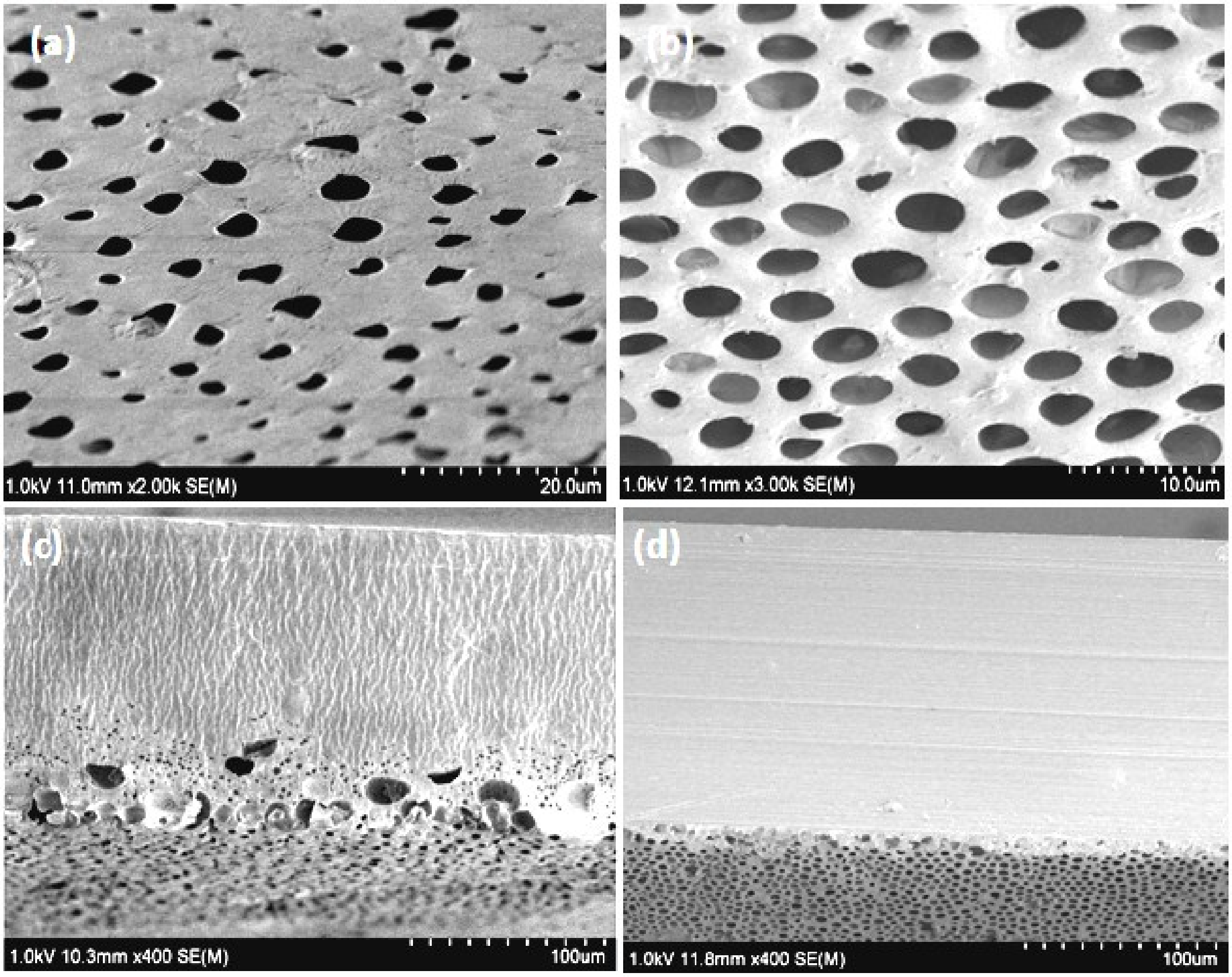

Figure SI 6. The SEM micrographs of the PMMA films obtained from solution of sample S_{4W10P} (a, c) and S_{4W15P} (b, d) where the PMMA concentration used was 10 (S_{4W10P}) and 15 (S_{4W15P}) wt % respectively. (a), (b) show the surface in contact with the Petri dish; (c), (d) are the corresponding cross sectional view. With increasing the polymer concentration porosity decreases and the thickness of the dense skin layer increases as shown in the cross sectional view (c and d).

Effect of initial casting solution thickness

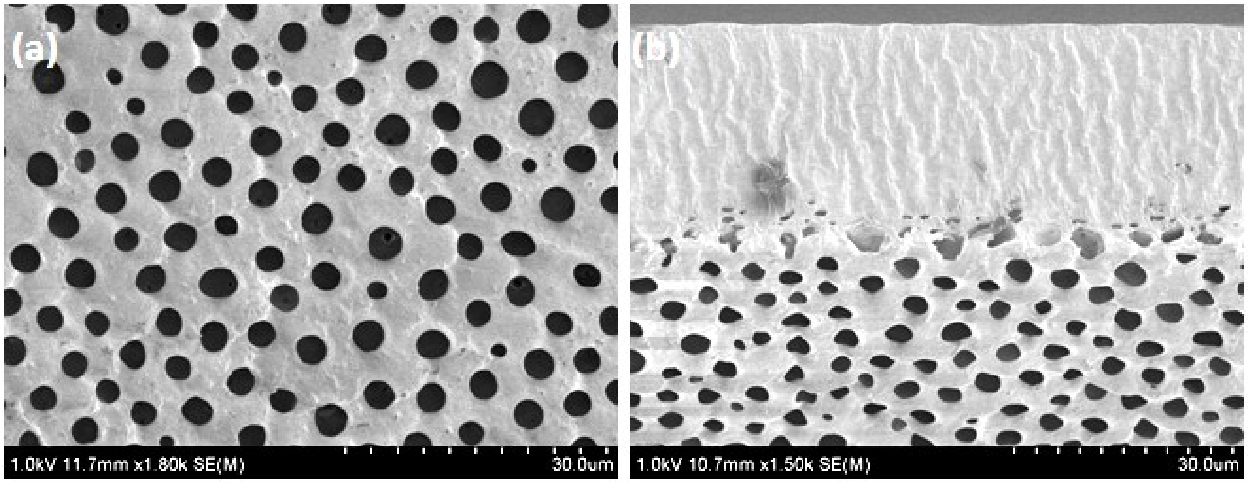

Figure SI 7. (a) The SEM micrographs of the PMMA film surface in contact with the surface of the Petri dish and (b) cross section obtained from casting solution corresponding to S_{3W5PTh} where initial casting solution thickness was 1mm. When the casting solution thickness was decreased, the porosity was also observed to decrease. Correspondingly, the dense skin layer thickness was also observed to increase compared to the film obtained from casting solutions of higher thickness (sample S_{3W5P}) despite similar experimental conditions.

Effect of relative humidity

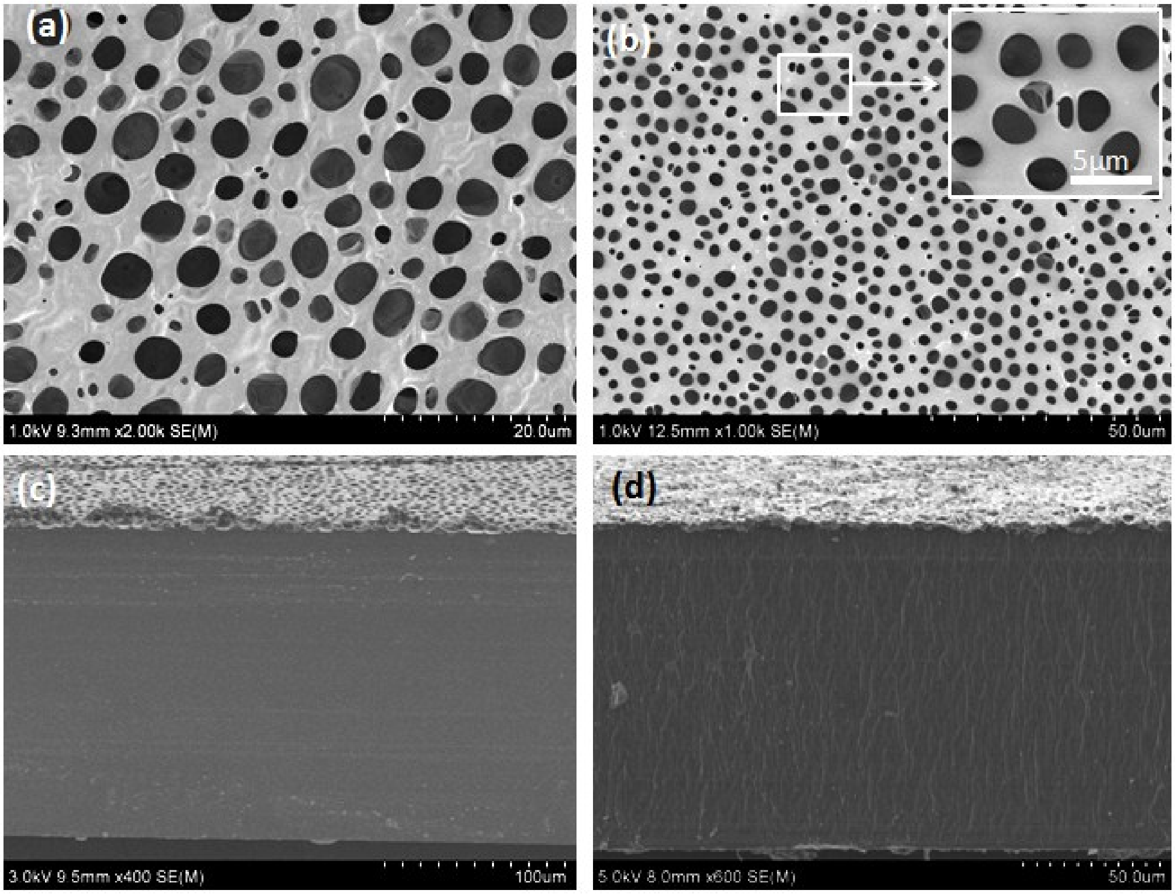

Figure SI 8. The SEM micrographs of the PMMA film top surface - (a), (b) and cross section view- (c), (d) obtained from evaporation of the casting solution corresponding to S_{0W5PH} and S_{1W5PH} respectively where relative humidity during the film formation was 74 %. At higher humidity (74%) the water drops were observed to condense on the air-solution interface, as a result of evaporative cooling effect. Therefore, as a result of higher water concentration at the top surface, the phase separation as well as the pores formation occurs at the top surface (i.e., at the air-solution interface).

Effect of evaporation temperature

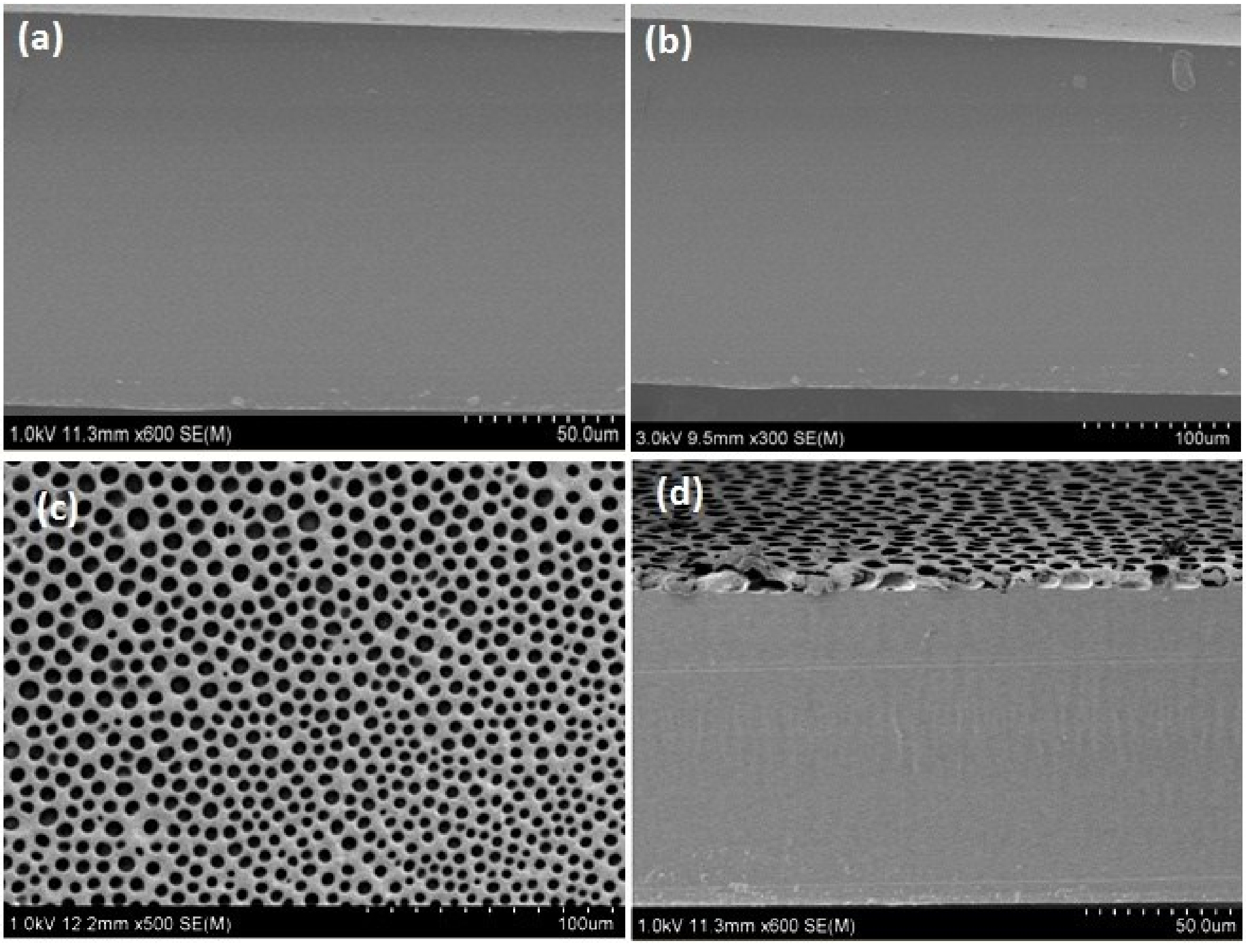

Figure SI 9. The SEM micrographs of the PMMA films - (a) and (b) are the cross section view of the film obtained from samples corresponding to S_{2W5PT} and S_{3W5PT}. (c) and (d) are top surface and cross section view respectively for the film obtained from S_{4W5PT} sample. These films were obtained by casting the solutions at 50°C. Both S_{2W5PT} and S_{3W5PT} show nonporous film while S_{4W5PT} shows only top surface pores. With increasing the temperature of the casting solution, the rate of evaporation of the solvent and the non solvent increases. Therefore both top and bottom surface composition does not reach phase boundary and hence nonporous films result from S_{2W5PT} and S_{3W5PT} samples. On the otherhand, for the sample S_{4W5PT}, the rate of evaporation of THF leads to a higher water concentration at the top surface and the composition reach the phase boundary as the initial water concentration (4 wt%) was more than S_{2W5PT} (2 wt%) and S_{3W5PT} (3 wt%). On the contrary, as water also evaporating at faster rate, the concentration of water inside the bulk of the casting solution was decreased. This results only top surface pore and nonporous bottom layer.

Characterization of PMMA films by FTIR and XRD analysis

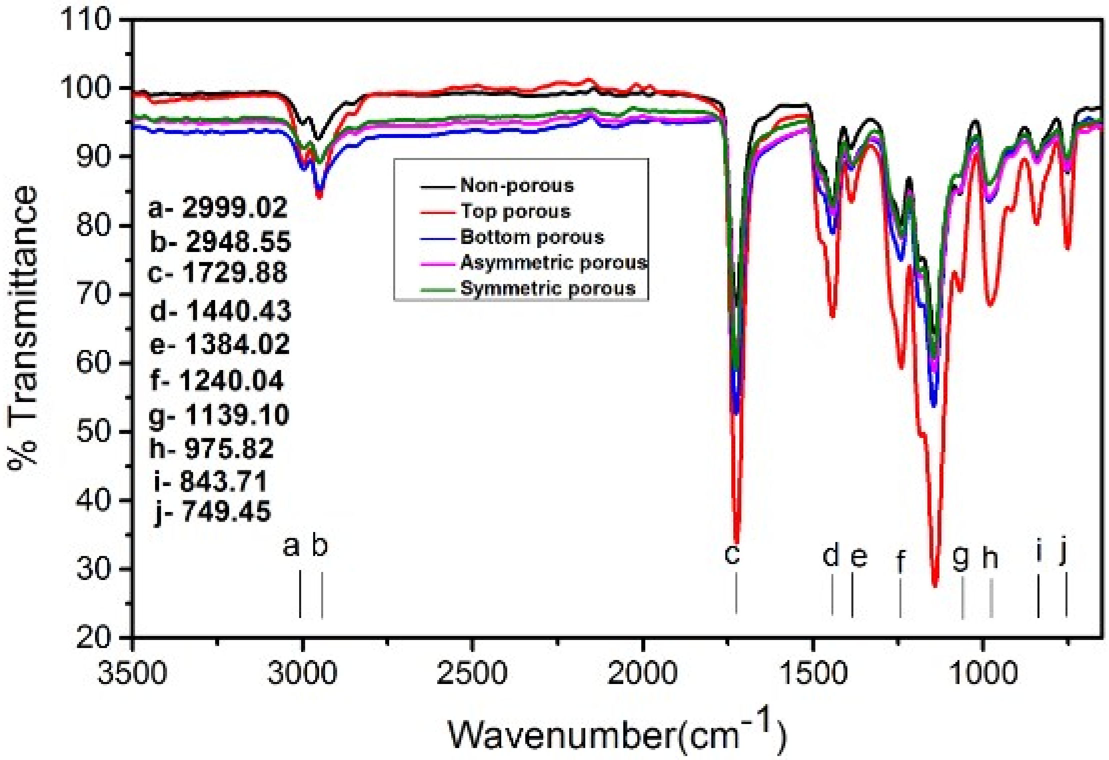

Figure SI 10. FTIR spectra of non-porous and porous PMMA films synthesized by evaporation induced phase separation method (EIPS)

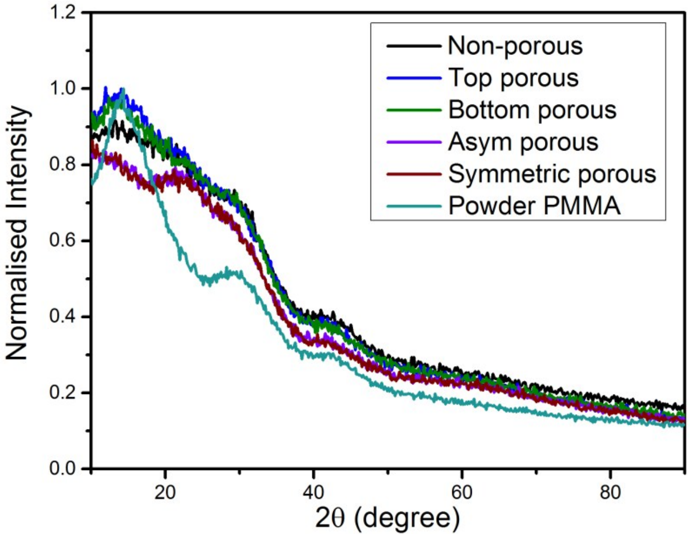

Figure SI 11. XRD spectra of PMMA films

Composition path of PMMA films

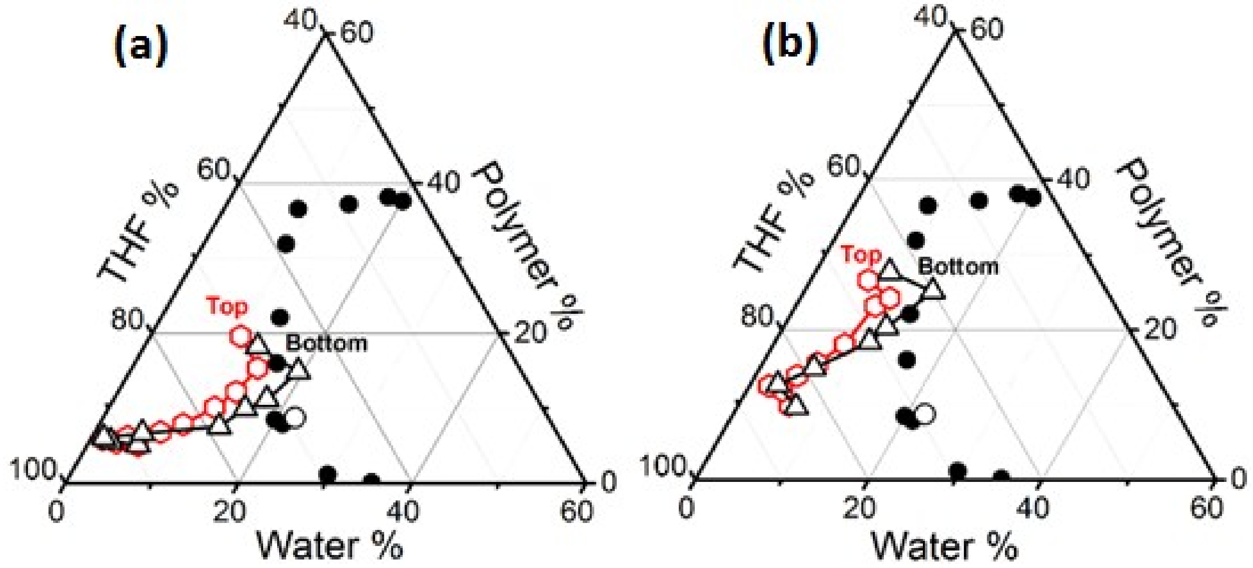

Figure SI 12. The composition paths (CP) for the casting solutions corresponding to the samples (a) S_{3W5P} and (b) S_{4W10P}. In both the cases, the top surface CP remains in the single phase region while bottom surface CP touches the phase boundary. The microstructures obtained from these casting solutions show more porous layer for S_{3W5P} than for S_{4W10P} as shown in Figure 3 d) and c) (in the manuscript). The films obtained from casting solution corresponding to S_{3W5P} and S_{4W10P} respectively showed an average porosity of 29.3% and 17.4 % even though water concentration is less for the former case. This may be attributed as the difference in the PMMA concentration in the casting solutions i.e., the PMMA concentration in S_{3W5P} was 5 wt %, lower than in S_{4W10P} (10 wt %). As PMMA concentration was higher for S_{4W10P}, the larger dense skin layer thickness was obtained from this casting solution. Also, the ratio water/PMMA at the point of phase separation was 1.5 for S_{3W5P}, higher than that for S_{4W10P} (0.6).
